# ATP and luciferase assays to determine the rate of drug action in *in vitro* cultures of *Plasmodium falciparum*

**DOI:** 10.1186/1475-2875-11-369

**Published:** 2012-11-07

**Authors:** Tasmiyah Khan, Anna C van Brummelen, Christopher J Parkinson, Heinrich C Hoppe

**Affiliations:** 1 CSIR Biosciences, PO Box 365, Pretoria, 0001, South Africa; 2 Department of Biochemistry, Microbiology & Biotechnology, Rhodes University, PO Box 94, Grahamstown, 1640, South Africa; 3 School of Biomedical Sciences, Charles Sturt University, PO Box 883, Orange, 2800, Australia

**Keywords:** *Plasmodium*, ATP, Luciferase, Chloroquine, Mefloquine, Artemisinin, Ritonavir, Gramicidin, Proteasome, DFMO

## Abstract

**Background:**

Knowledge of the rate of action of compounds against cultured malaria parasites is required to determine the optimal time-points for drug mode of action studies, as well as to predict likely *in vivo* parasite clearance rates in order to select optimal hit compounds for further development. In this study, changes in parasite ATP levels and transgenic luciferase reporter activity were explored as means to detect drug-induced stress in cultured parasites.

**Methods:**

*In vitro* cultures of *Plasmodium falciparum* 3D7 wild-type or firefly luciferase-expressing parasites were incubated with a panel of six anti-malarial compounds for 10 hours and parasite ATP levels or luciferase activity determined at two-hour intervals using luminescence-based reagents. For comparative purposes, parasite morphology changes were evaluated by light microscopy, as well as the extent to which parasites recover after 48 hours from a six-hour drug treatment using a parasite lactate dehydrogenase assay.

**Results:**

Changes in parasite ATP levels displayed three phenotypes: mild or no change (chloroquine, DFMO); 2–4 fold increase (mefloquine, artemisinin); severe depletion (ritonavir, gramicidin). The respective phenotypes and the rate at which they manifested correlated closely with the extent to which parasites recovered from a six-hour drug treatment (with the exception of chloroquine) and the appearance and severity of morphological changes observed by light microscopy. Luciferase activity decreased profoundly in parasites treated with mefloquine, artemisinin and ritonavir (34-67% decrease in 2 hours), while chloroquine and DFMO produced only mild changes over 10 hours. Gramicidin yielded intermediate decreases in luciferase activity.

**Conclusions:**

ATP levels and luciferase activity respond rapidly to incubation with anti-malarial drugs and provide quantitative read-outs to detect the appearance and magnitude of drug-induced stress in cultured parasites. The correlation between the observed changes and irreversible parasite toxicity is not yet sufficiently clear to predict clinical clearance rates, but may be useful for ranking compounds against each other and standard drugs *vis-à-vis* rate of action and for determining early time-points for drug mode of action studies.

## Background

The ability of the malaria parasite *Plasmodium falciparum* to develop resistance to drugs motivates the critical need to deliver new development candidates to bolster the current clinical pipeline [1]. Large-scale screens of synthetic chemical libraries have been especially successful in identifying numerous early hit compounds against cultured parasites [2]. Compared to these phenotypic screening programmes, target-based discovery approaches have been less successful, perhaps partially due to the lack of extensively validated drug targets. Further characterization of priority hit compounds may include an investigation of their modes of action, which could also yield new potential targets for malaria drug discovery. The mode of action of a compound may be assessed by determining the effect of the compound on specific biochemical or cell biological pathways, or by a more global approach, e.g. transcriptomic, proteomic and/or metabolomic profiling [3]. These studies require knowledge of the rate of action of a compound and should ideally be performed at early time-points when the compound starts exerting its primary effect(s), as opposed to later time-points when the primary mode of action may conceivably be obscured by non-specific secondary responses in the parasite. Moreover, a highly desirable property of anti-malarial compounds is that they should kill parasites rapidly, in order to reduce the required dosages in clinical use, minimize the likelihood of resistance development, and increase patient compliance. This requires an accurate determination of the rate of action of promising compounds against malaria parasites, particularly to enable researchers to rank compounds for further pre-clinical and clinical development.

A parasite reduction ratio (PRR) assay was recently described to predict the rate of parasite clearance *in vivo* by measuring the extent to which parasites recover from drug exposure for defined periods of time in *in vitro* cultures [4]. However, for mode of action studies, the rate of action is conventionally assessed by evaluating parasite morphological changes over time during drug exposure using Giemsa-stained blood smears and light microscopy. While this technique is relatively simple to perform, it is time-consuming, highly susceptible to subjective interpretation and difficult to express quantitatively, unless the morphological changes are drastic and uniform. Making the distinction between parasites with normal *vs* aberrant drug-induced morphologies is particularly challenging due to the heterogeneous morphology of individual parasites under routine culture conditions, the tendency of individual cells to display a spectrum of mild to severe morphological abnormalities, particularly at early time-points, and the challenge of preparing uniform microscopy preparations on separate occasions.

In this study, ATP quantitation and luciferase activity measurements as a means to detect the rate and severity of drug-induced stress in *in vitro* cultures of *Plasmodium falciparum* was explored. ATP content in cells as an indicator of metabolic status could conceivably be used to detect abnormal metabolic activity imposed by drug action, while luciferase activity in transgenic parasites was unexpectedly found to decrease rapidly and profoundly during drug exposure, which may be exploited as a novel indicator of the rate of drug-induced stress. The rate, magnitude and nature of the changes in parasite ATP content and luciferase activity levels over 10-hour incubation periods were characterized using a panel of six compounds with different modes of action: the clinical anti-malarial drugs chloroquine, mefloquine and artemisinin, and the experimental compounds DL-α-difluoromethyl ornithine (DFMO), an inhibitor of polyamine biosynthesis [5], ritonavir, an HIV aspartyl protease inhibitor with known anti-malarial activity [6] and gramicidin, a mixture of channel forming ionophores for monovalent cations [7].

## Methods

### Parasite cultivation, morphological evaluation and drug IC_50_ determination

*Plasmodium falciparum* 3D7 cultures were maintained at 37°C in medium consisting of RPMI 1640 supplemented with 2mM L-glutamine, 25mM Hepes, 20mM glucose, 0.65 mM hypoxanthine, 60 μg/mL gentamycin, 2.5% (w/v) Albumax II and 3% (v/v) type O^+^ red blood cells in flasks suffused with a mixture of 5% CO_2_, 5% O_2_, 90% N_2_. Parasite life-cycles were routinely synchronized by the sorbitol method [8]. Parasite morphology was assessed by light microscopy of methanol fixed and Giemsa-stained thin blood smears using a 100x oil-immersion objective. Images were captured with an Olympus BX41 upright microscope equipped with a CC12 Soft Imaging system. Drug 50% inhibitory concentrations (IC_50_) were determined by measuring parasite viability after a 48h incubation with 3-fold serial dilutions of the drugs using the parasite lactate dehydrogenase (pLDH) assay [9]. IC_50_ values were derived from non-linear regression dose–response plots prepared with GraphPad Prism (v.5.02).

### Drug compounds used in this study

Chloroquine diphosphate, mefloquine hydrochloride, artemisinin and gramicidin from *Bacillus brevis* were purchased from Sigma-Aldrich. DL-α-difluoromethylornithine (DFMO) was kindly provided by P. Woster (Wayne State University, MI) and ritonavir purchased from Kinbester Co. (Xiamen, China). The compounds were prepared as 10mM stock solutions in DMSO (artemisinin, gramicidin, ritonavir), methanol (mefloquine) or water (chloroquine, DFMO). The proteasome inhibitors lactacystin and MG-132 were purchased from Merck and prepared as 10mM stocks in water and DMSO, respectively. Final concentrations of the drug compounds used in all assays in this study were: 100 nM chloroquine; 100 nM mefloquine; 100 nM artemisinin (ATP, morphology and recovery assays); 500 nM artemisinin (luciferase assay); 0.1 nM gramicidin; 10 mM DFMO; 100 μM ritonavir; 5 μM lactacystin; 300 nM MG-132.

### Parasite recovery assay

An early trophozoite-stage culture was used to prepare a 2% haematocrit, 2% parasitaemia suspension in culture medium which was distributed into 6-well plates at 2.5 mL per well. The plates were treated with drug and solvent control solutions, each in triplicate. An additional plate was prepared with uninfected RBCs in triplicate wells to serve as a background control. The plates were transferred to an airtight chamber suffused with 5% CO_2_, 5% O_2_, 90% N_2_ and incubated at 37°C for six hours. Following incubation, the contents of each well was mixed well and 800 μL was transferred to a sterile microfuge tube to pellet the infected red blood cells. The pellet was washed three times in 1 mL of culture medium pre-warmed at 37°C and then resuspended in fresh medium at a haematocrit of 1%. The suspension was transferred to a 96-well plates at 200 μL per well. Parasite lactate dehydrogenase activity [9] was measured in the plate after it was returned to the airtight chamber, gassed and incubated at 37°C for 48 hours. The pLDH activity was used to calculate the percentage parasite viability after 48h for each respective drug, relative to solvent controls.

### ATP assay

To quantitate changes in parasite ATP content during drug exposure, early trophozoite-stage cultures were used to prepare a 32 mL 5% haematocrit, 2% parasitaemia suspension in culture medium. The suspension was split into 2 x 15 mL cultures and incubated in culture flasks at 37°C with drug compounds and solvent control solutions, respectively. Three 500 μL aliquots of the culture suspensions were removed from the control and treated cultures, respectively, every 2 hours over a 10-hour period and transferred to cold microfuge tubes placed on ice. The infected red blood cells in the 500 μL samples were pelleted by centrifugation at 8,000 rpm for 30 seconds in a microfuge and lysed by adding 500 μL 0.24% (w/v) saponin, 0.1% (w/v) bovine serum albumin (BSA) in phosphate-buffered saline (PBS). Complete RBC lysis was achieved by vortexing the solution for approximately 45 seconds until translucent. The lysate was removed and pipetted onto the surface of a 300 μL mixture of dibutyl phthalate and dioctyl phthalate (5:4) in a microfuge tube and centrifuged at 14,000 rpm for 40 seconds. This resulted in the intact parasites pelleting at the bottom of the tube, while the aqueous RBC lysate settled above the intervening phthalate oil layer. The aqueous layer was aspirated off and the inner surface of the tube above the oil layer and the top of the oil layer carefully washed with 500 μL of 0.1% (w/v) BSA in PBS to completely remove all traces of RBC lysate. The oil layer was aspirated off, 150 μL ice-cold PBS added onto the parasite pellet and the sample snap-frozen in liquid nitrogen and stored at −20°C. ATP levels were subsequently measured by thawing the samples at room temperature, resuspending the parasite pellets by pipetting, transferring 50 μL to a white 96-well plate and adding 50 μL of CellTitre-Glo® reagent (Promega). The plate was briefly agitated and then incubated in the dark for 10 minutes at room temperature before measuring luminescence in Tecan Infinite F500 plate reader. Average background luminescence readings from wells containing PBS alone were subtracted from the sample readings.

### Preparation of luciferase transgenic parasites

To obtain an expression plasmid for stable episomal expression of luciferase, the expression and selection cassettes of pHTK were sub-cloned into the *NotI* and *NcoI* sites of pGEM-T Easy (Promega). The pHTK expression cassette consists of the *P. falciparum* heat-shock protein *hsp86* 5’-untranslated promoter region, the *Herpes simplex* virus thymidine kinase coding sequence flanked by *XhoI* sites and the *P. berghei* 3’ termination region, while the selection cassette contains the human dihydrofolate reductase (*hdhfr*) coding sequence flanked by the *P. falciparum* calmodulin 5’-untranslated promoter region and the *P. falciparum* histidine-rich protein 2 3’-untranslated region in a head-to-head orientation with the expression cassette [10]. The coding sequence of the *Photinus pyralis* luciferase gene, flanked by *NheI* and *XhoI* restriction sites, was PCR amplified from the pGL2 plasmid (Promega) and replaced the thymidine kinase sequence in the pHTK expression cassette to obtain pHsp-Luc. The plasmid was used to transfect *P. falciparum* 3D7 parasites by electroporation (0.31 kV, 950μF)in a BioRad Gene-Pulser electroporator and stable lines selected by culturing in medium supplemented with 2.5 nM WR99210 according to previously described protocols [11].

### Luciferase assay

Transgenic luciferase-expressing parasite cultures at the early trophozoite stage were used to prepare 5% haematocrit, 2% parasitaemia suspensions in culture medium and 200 μL transferred to wells in a 96-well culture plate. A separate plate was prepared for each 2-hour time-point of the assay. Test drug compounds and solvent control solutions were added to triplicate wells in the plate, while uninfected RBCs at 5% haematocrit (200 μL) were added to triplicate wells as background controls. The plates were transferred to an airtight chamber suffused with 5% CO_2_, 5% O_2_, balance N_2_ and incubated at 37°C. At 2-hour intervals, one plate was carefully removed from the chamber without disturbing the settled RBCs and 150 μL of supernatant was removed from all wells, followed by the addition of 100 μL per well of Glo® Lysis Buffer (Promega). After 5 minutes incubation at room temperature, the contents of the wells were mixed by pipetting, 100 μL transferred to a white 96-well plate 100 μL of Bright-Glo® Luciferase Assay System reagent (Promega) added and the luminescence was measured immediately in a Tecan Infinite F500 multimode plate reader. The average luminescence readings obtained from uninfected red blood cell wells were subtracted as background from those obtained with drug-treated and untreated infected red blood cell cultures.

## Results

### Drug-induced changes in parasite ATP levels

An assay format based on the use of the luminescent CellTitre-Glo® reagent (Promega) was developed for determining changes in parasite ATP levels. Briefly, aliquots were removed from test and control cultures and transferred to microfuge tubes. To remove extraneous RBC-associated ATP, the cells were lysed in saponin and the parasites separated from the lysate by centrifugation through a phthalate oil layer. The parasite pellets were resuspended in CellTitre-Glo® reagent, transferred to microtitre plates and luminescence read in a multimode plate reader. In trophozoite-stage cultures, the assay routinely yielded luminescence signals more than 250-fold higher than those obtained with uninfected RBC controls, producing Z’-factor values [12] of 0.95-0.97, and luminescence correlated linearly with parasite numbers over the range 1x10^5^ – 2x10^6^ ( Additional file 1).

**Figure 1:**
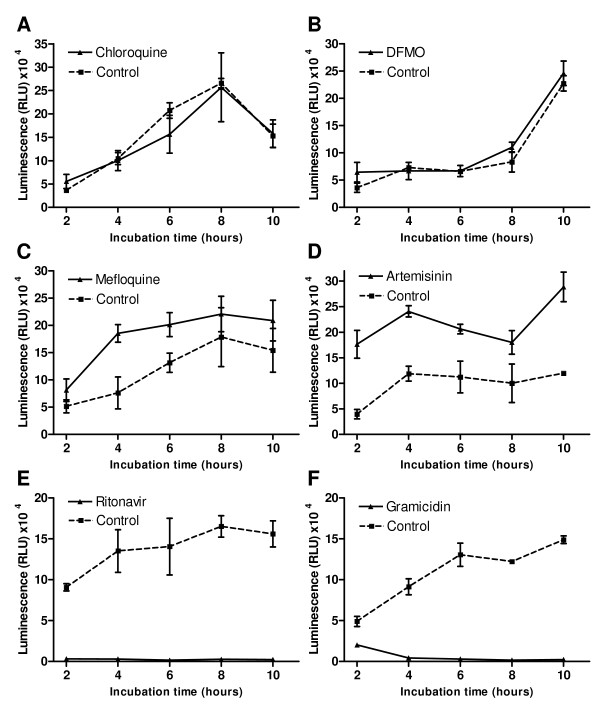
**Variation in ATP levels in drug-treated parasites. ***Plasmodium falciparum* 3D7 parasite cultures were incubated for 10 hours with 100 nM chloroquine (**A**), 10 mM DFMO (**B**), 100 nM mefloquine (**C**), 100 nM artemisinin (**D**), 100 μM ritonavir (**E**) or 0.1 nM gramicidin (**F**). At 2-hour intervals, aliquots were removed from drug exposed (solid line) and solvent control (dotted line) cultures, parasites were isolated by saponin lysis and centrifugation through a phthalate oil layer, and their ATP content determined with a luminescence based CellTitre-Glo® reagent (Promega). Individual data-points are mean luminescence values (relative light units – RLU) from three aliquots per culture processed in parallel and error bars indicate the standard deviation.

To determine if parasite ATP levels change in response to drug-induced stress, and the dimensions and rates of the changes, early trophozoite-stage cultures were incubated with a panel of six anti-malarial drug compounds and aliquots were removed at 2-hour intervals over a 10-hour period to quantify ATP content relative to untreated controls (Figure 1). Compound concentrations used were approximately 5 times their IC50s determined using the parasite lactate dehydrogenase assay and published data ( Additional file 2). In parasites exposed to chloroquine and DFMO (Figures 1A and 1B, respectively), ATP levels matched those of untreated control parasites over the entire 10-hour incubation period (t-test P values >0.05 at all time-points). Note that the ATP levels in control parasites fluctuate extensively during the trophozoite stage ( Additional file 3). By contrast, in mefloquine-treated parasites (Figure 1C) there was a marked 2.4-fold increase in ATP by the 4h time-point and ATP levels remained consistently elevated above those of controls during the remainder of the 10-hour incubation, although the 8h and 10h luminescence values display t-test P values >0.05 compared to the controls. A more profound and rapid increase in ATP content was observed during artemisinin treatment (Figure 1D). ATP content was 4.5-fold higher than those of untreated parasites at the 2h time-point and remained raised 1.8 to 2.4-fold during the rest of the 10-hour incubation (P<0.05 at all time-points). In marked contrast, ritonavir and gramicidin treatment caused a rapid and sharp decrease in parasite ATP levels (Figures 1E and 1F). Parasite ATP content was essentially depleted (luminescence <5% relative to controls) at the 2h and 4h time-points after addition of ritonavir and gramicidin, respectively.

**Figure 2:**
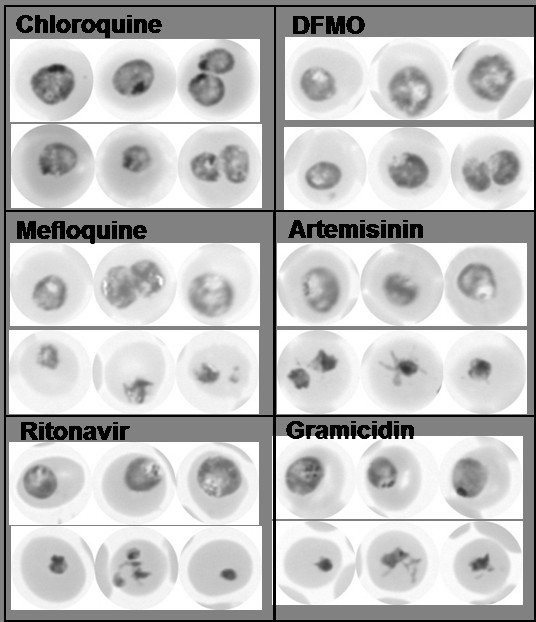
**Parasite morphology after 6–8 hours of drug treatment. ***Plasmodium falciparum* 3D7 parasite cultures were incubated for 8 hours with 100 nM chloroquine or 10 mM DFMO, or for 6 hours with 100 nM mefloquine, 100 nM artemisinin, 100 μM ritonavir or 0.1 nM gramicidin. Giemsa-stained thin blood smears were prepared from drug-exposed and solvent control cultures and viewed by bright-field light microscopy using a 100x oil-immersion objective. In each case, three representative images of control (upper row) and treated (lower row) parasites are shown.

To assess whether the observed responses of parasite ATP levels to drug exposure corresponded with morphological changes, Giemsa-stained smears were prepared at all time-points during the incubations and viewed by light microscopy (representative parasite images at the 8h time-point for chloroquine and DFMO and 6h time-point for the remaining drugs shown in Figure 2). Treatment with chloroquine and DFMO did not significantly alter parasite ATP levels and also produced the mildest morphological changes during the 10-hour incubation. Treated and control parasite morphologies were indistinguishable over most of the incubation period (Figure 2). A modest reduction in parasite size could be discerned at the 8h and 10h time-points, perhaps suggestive of slower parasite growth (i.e. cell cycle delay). In the mefloquine-treated parasite population, marked evidence of reduced parasite growth (i.e. smaller size) was observed at the 6h time-point (Figure 2), becoming more prominent later. Irregularly shaped and pyknotic (highly shrunken and stained deep purple) parasites were also observed at later time-points, but these represented a minority of the parasite population. Artemisinin treatment produced similar morphological changes to those accompanying mefloquine treatment, although irregularly shaped parasites were observed earlier, at 6h (examples shown in Figure 2). Ritonavir and gramicidin rapidly depleted parasite ATP levels and also produced the most rapid and significant morphological deterioration of the parasites. In ritonavir-treated cultures, a reduction in parasite size, abnormal morphologies and pyknotic cells were prevalent at 4h, and the latter predominated at later time-points (Figure 2). The same morphological changes were observed with gramicidin-treated parasites, with a preponderance of pyknotic (presumably dead) parasites at 6h (Figure 2).

**Figure 3:**
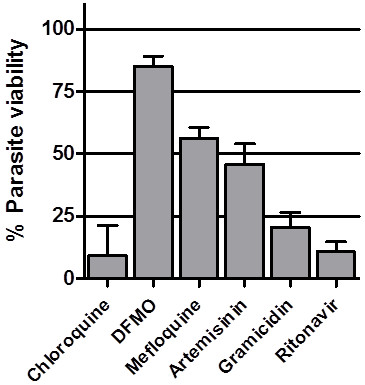
**Parasite recovery from a 6-hour drug exposure. ***Plasmodium falciparum* 3D7 parasite cultures were incubated for 6 hours with 100 nM chloroquine, 10 mM DFMO, 100 nM mefloquine, 100 nM artemisinin, 100 μM ritonavir or 0.1 nM gramicidin. The infected erythrocytes were washed and returned to culture in drug-free medium for an additional 48 hours, after which pLDH activity was determined for each culture (solid bars). Percentage parasite viability was calculated from pLDH activity relative to solvent-treated controls after subtraction of background values obtained with uninfected control erythrocyte cultures. Each drug treatment was performed in three parallel cultures and the bars represent the mean percentage parasite viability values and the error bars standard deviation.

Conceivably, increased ATP levels observed with mefloquine and artemisinin may represent a parasite metabolic response to cope with drug-induced stress, while the marked reduction in ATP observed with ritonavir and gramicidin indicates a serious deterioration in parasite metabolism. The question arose to what extent these changes reflect a terminal compromise in parasite viability. To address this, a short “parasite recovery assay” was performed. Briefly, parasite cultures were incubated with the respective drug compounds for 6h, after which the compounds were removed by washing and the treated parasites returned to culture for an additional 48h. After the 48h incubation, parasite levels were determined by measuring parasite lactate dehydrogenase (pLDH) activity and expressed as % parasite viability relative to untreated controls (Figure 3). Consistent with the modest ATP and morphological changes observed previously with DFMO treatment, parasites were able to recover effectively from the 6h exposure to DFMO and achieved 85% parasite viability. Mefloquine and artemisinin treatment for 6h resulted in a more irreversible loss of parasite viability and parasite levels of 56% and 46% relative to controls were obtained for the two drugs, respectively. By comparison, only 11% and 21% of ritonavir- and gramicidin-treated parasites, respectively, recovered from the 6-hour drug treatment, compared to untreated controls. Unexpectedly, considering the mild ATP and morphological changes previously observed with chloroquine, only 9% of parasites recovered from the 6h chloroquine treatment. Conceivably, this is due to the irreversible accumulation of chloroquine in the parasite food vacuole due to pH trapping [13], which effectively counteracts removal of the drug from exposed parasites by washing. However, it has been reported that chloroquine may be effectively washed out of treated parasites [14]. An alternative explanation could be that free heme that accumulates in the parasite due to chloroquine action in the 6h incubation [15] remains associated with parasite membranes and continues to exert toxicity during the subsequent 48h after chloroquine removal. 

### Drug-induced changes in transgenic parasite luciferase levels

**Figure 4:**
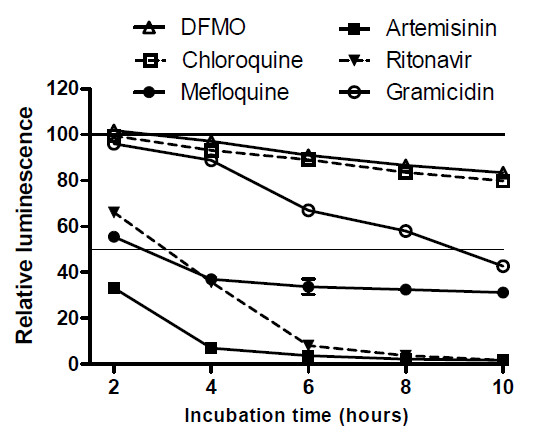
**Variation in luciferase activity in drug-treated transgenic parasites. ***Plasmodium falciparum* 3D7 parasites expressing firefly luciferase were incubated for 10 hours with chloroquine, DFMO , mefloquine, artemisinin , ritonavir or gramicidin in 96-well plates. At 2-hour intervals, luciferase activity in drug-exposed and solvent control cultures was determined with a luminescence based Bright-Glo® Luciferase Assay System reagent (Promega). At each time-point, luminescence is plotted as a percentage relative to that obtained with controls incubated with solvent alone, after background luminescence produced by uninfected red blood cells had been subtracted. Individual data-points are the mean relative luminescence values from three replicate wells and error bars indicate standard deviation.

While assessing the effect of anti-malarial compounds on transgenic parasites expressing luciferase, it was unexpectedly found that luciferase activity markedly and rapidly decreased soon after drug exposure. To explore if this phenomenon extends to the six drug panel used in the ATP and other assays described above and the extent to which the luciferase activity changes correlate with the previous results, cultures containing parasites stably expressing firefly luciferase were incubated with the respective compounds for 10 hours and luciferase activity determined at 2h intervals using the luminescent Bright-Glo® luciferase assay reagent (Promega). With chloroquine and DFMO treatment, there was only a modest and gradual decline in luciferase activity in treated parasites (Figure 4). By the 10h time-point, luciferase activity had decreased by 20% and 17% in chloroquine and DFMO treated parasites, respectively. By contrast, artemisinin, mefloquine and ritonavir produced rapid and profound reductions in luciferase activity. Luciferase activity had decreased by 67%, 44% and 34% after only 2h treatments with artemisinin, mefloquine and ritonavir respectively. Luciferase activity had been virtually abolished (93% decrease) after 4h of artemisinin treatment and 6h of ritonavir treatment (92% decrease; Figure 4). The luciferase activity decrease was less severe in the mefloquine treated parasites and activity remained at 37% - 31% of the control levels during the 4–10 hour period. Gramicidin treatment produced an intermediate decrease in luciferase activity. Activity had decreased by 33% at the 6h time-point and 43% at 10h.

**Figure 5:**
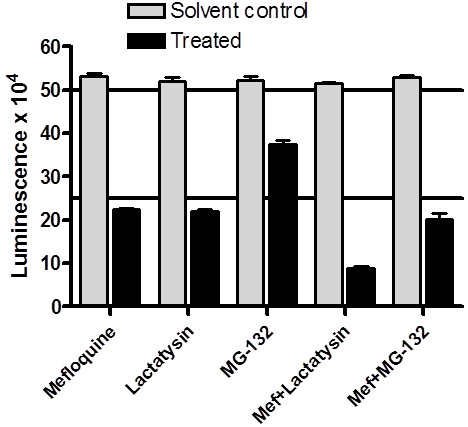
**Effect of proteasome inhibitors on mefloquine-induced luciferase activity decrease. ***Plasmodium falciparum* 3D7 parasites expressing firefly luciferase were incubated for 6 hours with 100 nM mefloquine, 5 μM lactacystin or 300 nM MG-132, or with 100 nM mefloquine combined with lactacystin or MG-132 in a 96-well plate. Luciferase activity in drug-exposed (solid bars) and solvent control (shaded bars) cultures was determined with a luminescence based Bright-Glo® Luciferase Assay System reagent (Promega). Individual data-points are mean luminescence values (relative light units – RLU) from three replicate wells and error bars indicate standard deviation.

To determine if the rapid reduction in luciferase activity is due to proteasomal digestion as a drug stress response, the effect of two proteasome inhibitors, lactacystin and MG-132 [16], on drug-induced luciferase activity reduction was assessed. Treatment concentrations with the two inhibitors were based on their respective IC50s ( Additional file 2). Parasites were incubated for 6h in medium containing respectively mefloquine, lactacystin or MG-132, or mefloquine in combination with lactacystin or MG-132, and parasite luciferase levels determined (Figure 5). As expected, mefloquine treatment for 6h caused a 58% decrease in luciferase activity. However, both lactacystin and MG-132 alone also markedly decreased luciferase activity (by 58% and 28% respectively) and this effect was further exacerbated by co-incubation with mefloquine. This suggests that proteasome degradation is not responsible for the luciferase activity reduction and, moreover, that the decrease in luciferase levels also extends to the two proteasomal inhibitors and may be a general parasite response to drug exposure. 

## Discussion

In this study, two assay formats as a means for determining the timing and severity of drug-induced stress in *Plasmodium falciparum* parasite cultures over short incubation periods were explored. A panel of six anti-malarial drug compounds with different modes and, presumably, rates of action were used to interrogate and characterize the utility of the ATP and luciferase assays described above. The observed responses of trophozoite-stage parasite ATP content to drug exposure over a 10 hour incubation period can be broadly classified into three phenotypes: little or no change relative to untreated controls (chloroquine and DFMO); marked and sustained increase in ATP levels (mefloquine and artemisinin); rapid depletion of ATP content (ritonavir and gramicidin). These classifications may conceivably be interpreted as signifying, respectively: absence of significant drug-induced stress; altered (increased) metabolic activity to counteract drug-induced stress; severely compromised metabolic status due to drug action. This interpretation correlates with the results obtained with subsidiary assays. Morphological evaluation of the drug-treated parasites revealed mild abnormalities, mostly limited to growth retardation, in the ATP non-responders, and (with the exception of chloroquine) competence to recover from a 6h drug exposure. By contrast, increased ATP levels correlated with earlier appearance of growth-inhibited parasites and additional aberrant morphologies and a 44%-54% reduction in recovery following 6h drug exposure, while rapid ATP depletion was accompanied by the early appearance and preponderance of pyknotic (presumably irretrievably compromised) parasite forms and a significantly greater inhibition (79%-89%) of parasite recovery following 6h drug exposure. Interestingly, the timing and magnitude of the ATP changes, whether increased or decreased, also correlated with the timing of appearance of abnormal morphologies and ability to recover from brief drug exposure, although arguably more compounds will need to be assessed to determine the validity of this trend. Based on these results, the rate and severity of drug stress can be tentatively ranked as ritonavir>gramicidin>artemisinin>mefloquine>>chloroquine, DFMO.

The lack of a notable ATP response with DFMO agrees with its accepted mode of action, which is cytostatic rather than cytocidal. It inhibits ornithine decarboxylase, a key enzyme in polyamine biosynthesis, which slows down growth and eventually blocks parasite cell cycle progression in the late trophozoite stage in a manner which is reversible by exogenous addition of polyamines to the medium [5]. Thus, a 10h incubation with DFMO starting in the early trophozoite stage may generate insufficient stress to result in a notable disruption of ATP homeostasis. The consensus view is that chloroquine becomes ionized and trapped in the low pH environment of the parasite food vacuole, where it disrupts haemozoin formation and causes an accumulation of toxic free haem and chloroquine-haem complexes [17]. The results of this study suggest that a 10h incubation with chloroquine from the early trophozoite stage does not generate sufficient haem complexes to exert a significant effect on parasite ATP levels and/or haem-induced toxicity is slow-acting. Interestingly, the failure of parasites to recover from the 6h chloroquine incubation in the recovery assay may provide additional evidence for the irreversible entrapment of chloroquine in the food vacuole, where it probably continues to cause haem accumulation and toxicity despite the washing away of exogeneous chloroquine in the medium. Thus, the trapping phenomenon likely contributes greatly to the effectiveness of chloroquine as an anti-malarial drug, despite the apparently slow rate at which it induces metabolic stress compared to the other compounds. An alternative explanation is that haem accumulating in the parasite during the 6h chloroquine treatment remains parasite associated after chloroquine washout and continues to exert toxicity in the following 48h incubation.

Despite decades of clinical use, the mode of action of mefloquine is still uncertain. A general assumption is that it shares chloroquines effect on haemozoin formation and causes a toxic accumulation of haem or haem complexes [18]. However, cell biological evidence e.g. the differential effects of the two drugs on haemoglobin endocytosis, trafficking and digestion suggests otherwise [19,20]. In the present study, the rapid and marked effect of mefloquine on ATP levels and luciferase activity is not mirrored by chloroquine and thus strongly implies a different primary mode of action for mefloquine. As for mefloquine, the mode of action of artemisinin is uncertain and has been suggested to involve *inter alia* inhibition of the parasite equivalent of the sarcoplasmic reticulum Ca^2+^-ATPase (PfATP6), depolarization of mitochondrial membranes, haem adduct formation, generation of reactive oxygen species in conjunction with haem and protein alkylation [21]. Nonetheless, the appeal of artemisinin as an anti-malarial is partly based on its perceived rapid action against parasites [4], which is supported by the observations from this study that ATP and luciferase activity levels in treated parasites are markedly affected by the drug at the first 2h time-point of exposure. Uncertainty regarding the modes of action of mefloquine and artemisinin make it difficult to fully explain the cause for increased ATP levels in treated parasites, other than to make a general assumption that it reflects increased metabolic activity by the parasite as part of a cellular stress response to overcome detrimental drug effects. This likely necessitates increased production of ATP to fuel synthesis and activities of enzymes, substrates and co-factors involved in e.g. antioxidant defence and protein chaperone systems.

The very rapid and profound depletion of ATP in ritonavir-treated parasites was supported by the early preponderance of pyknotic parasite morphologies and highly compromised ability to recover from a 6h drug exposure. This was surprising, given that ritonavir is an HIV protease inhibitor and was proposed to act against parasites by inhibiting aspartyl proteases responsible for haemoglobin digestion [22]. Arguably, inhibition of this process would result in a more protracted growth inhibition of parasites due to amino acid starvation, not the rapid and lethal effect observed here. This argues for a different mode of action of ritonavir, which was also proposed in a study reporting the anti-malarial interactions of HIV protease inhibitors with hemoglobin protease inhibitors, mefloquine and chloroquine [23]. The rapid depletion of parasite ATP by gramicidin, however, is consistent with its probable mode of action. Gramicidins are lipophilic, linear peptides that form channels in membranes that are permeable to monovalent cations [24]. The rapid disruption of cellular sodium, potassium and proton gradients through these channels should have immediate pleiotropic consequences for parasite metabolism, which may also be reflected by the extreme potency of gramicidin against parasites (IC_50_ < 0.1nM – Additional file 2).

The results obtained with the ATP assay suggest that it could represent a sensitive, quantitative means for detecting the earliest time-points of drug-induced stress to inform and complement drug mode of action studies. However, the question remains whether it could also be a useful tool for unambiguously determining the rate and extent to which parasite viability is irrevocably compromised by a particular drug. In principle, a total depletion of ATP could have been regarded as a signpost for irreversible parasite lethality. However, this is not entirely the case, as evidenced by the ability of ritonavir and gramicidin-treated parasites to recover from a 6h treatment, albeit severely limited, despite an apparent complete reduction in ATP in 2–4 hours. Conversely, artemisinin and mefloquine treated parasites actually display increased ATP levels at 6h, despite the fact that their recovery from a 6h treatment is inhibited by approximately 50%. The fact that treatment with the panel of six drugs produces three distinct phenotypes of ATP responses (increased, decreased and unchanged ATP levels) may further complicate a detailed interpretation of ATP responses to experimental drug pressure, necessitating an exploration of ATP responses with a larger drug panel before considering scale-up of the assay. In addition, 5xIC_50_ drug concentrations were used in this study as a compromise to obtain sufficient drug pressure on the parasite without excessive off-target effects unrelated to the primary drug mode of action which may be prevalent at higher concentrations. However, it should be cautioned that 5xIC50 does not necessarily equate to lethal dose to the same extent for all compounds. Therefore, rate of killing studies may best be performed at lethal dose concentrations. Characterization of the assay, perhaps in conjunction with the recently described parasite recovery rate (PRR) assay [4], would be required to more firmly define the correlates of ATP levels and irreversible parasite lethality. Nonetheless, the proposed ranking of the test drugs based on ATP responses, as discussed above, suggests that the assay in its current form may be used to assess the rate of parasite viability inhibition of experimental compounds relative to each other and standard “benchmark” drugs.

Serendipitously, it was found that luciferase activity in transgenic parasites responds rapidly and markedly to drug exposure. A highly attractive advantage of the luciferase assay is that it is entirely multiwell plate-based, requires minimal liquid handling steps and provides an extremely sensitive and robust read-out, thus making it potentially amenable to high-throughput formats. The overall trend in the results was similar to that obtained with the ATP assay. DFMO and chloroquine produced a slow, mild decrease in luciferase activity, while artemisinin, mefloquine and ritonavir profoundly compromised luciferase activity within 2 hours. The rapid decreases in luciferase activity during drug exposure could be construed as a cellular stress response in which proteolysis and amino acid release and/or selective translational inhibition is used to alter the proteome of the parasite [25,26]. Luciferase is particularly known to be susceptible to proteolytic degradation [27]. Nonetheless, the activity decrease was not affected by proteasome inhibitors, even though proteasomes are principally responsible for cytoplasmic protein turnover and homeostasis in mammalian cells [26]. Interestingly, the proteasome inhibitors on their own also produced marked loss of luciferase activity in 6 hours, suggestive of parasite stress experienced by the inhibition of their protein turnover ability. The rapid loss of luciferase activity is also not shared by all parasite cytoplasmic proteins. In contrast to luciferase, parasite pLDH activity showed only mild changes after 6 hours incubation with all the drugs, which hints that luciferase is particularly sensitive to cellular stress conditions. Caution should be exercised in interpreting the luciferase results, however. The luciferase assay requires the overexpression of a foreign reporter protein in the parasite. Conceivably, this could cause subtle alterations that may obscure drug-specific effects in subsequent drug mode of action studies (e.g. by transcriptomic, proteomic or metabolomic analyses). In addition, transgene overexpression might alter parasite sensitivity and the rate of drug-induced stress by certain compounds. For example, the detailed ranking of compound rate of action in the luciferase assay would differ from that obtained by the ATP assay. Artemisinin produced more profound and rapid changes in luciferase activity than the other drugs, while gramicidin had a milder effect than expected from the earlier assays (ATP, morphology and parasite recovery). While mindful of these caveats, the ease of the assay still suggests that it could be used as a preliminary screen for rapid vs. slow acting compounds over a 4–6 hour incubation period, especially when a large number of compounds need to be assessed.

## Conclusion

The magnitude, nature and rate of changes in ATP levels in parasites incubated with drug compounds appear to correlate well with the severity and rate of drug-induced parasite stress, as judged by the recovery of parasites from a brief drug exposure and morphological changes. It suggests that the assay may be used to detect the early time-points of drug action for mode of action studies and to rank the rate of parasite viability inhibition of experimental compounds relative to each other and conventional drug standards. Luciferase activity in transgenic parasites decreased profoundly and rapidly during drug exposure at rates broadly comparable to those observed in the ATP assay. While correlation with parasite recovery and morphological changes were not as conclusive as that obtained with ATP, it may be useful as an initial screening tool to differentiate between rapid and slow acting compounds.

## Competing interests

The authors declare that they have no competing interests.

## Authors’ contributions

HCH and CJP conceived and designed the study, TK and ACvB performed the experimental procedures and data analysis, HCH and CJP were principally responsible for interpretation of the data. HCH and TK drafted the manuscript, which was read, amended and approved by all authors. All authors read and approved the final manuscript.

## Supplementary Material

Additional file 1 **Figure A1.** Correlation of ATP luminescence signals with parasite numbers and stages. A. Aliquots were removed from a trophozoite-stage culture and the parasites isolated. Serial dilutions of the parasites were performed in PBS and ATP levels measured. Parasite numbers were calculated from the percentage parasitaemia and red blood cell concentration (determined with a haemocytometer) of the original culture. Three samples were processed in parallel. Note that the lowest parasite number (1.23x10^5^) corresponds to approximately 4μl of a 2% parasitaemia, 5% haematocrit culture (or 0.2μl packed red blood cells) and produced an average luminescence reading of 7030. B. Ring-stage parasites were isolated from three 0.5ml aliquots of a sorbitol synchronized 5% haematocrit 10% parasitaemia culture and snap-frozen in liquid nitrogen. After 24h, additional 0.5ml aliquots were removed from the same culture and used to prepare frozen trophozoite-stage parasites. After thawing, ATP luminescence signals in the samples were determined and compared to background signals obtained with aliquots of uninfected red blood cell cultures processed in parallel. The trophozoite samples produced a mean luminescence reading of 354054, compared 13121 for rings and 993 for uninfected red blood cells (note the log scale of the Y-axis). Click here for file

Additional file 2 **Table 1.** IC50 values of compounds used in this study. IC50 values were obtained by incubating *P. falciparum* 3D7 cultures with serial dilutions of the test compounds for 48 hours and assessing parasite viability using the parasite lactate dehydrogenase (pLDH) assay. Values are shown as averages ± standard deviation for three independent determinations. IC50 values for lactacystin and MG-132 were determined on a single occasion against luciferase-expressing parasites. Click here for file

Additional file 3 **Figure A2.** ATP changes during trophozoite development. A tightly synchronized culture of *Plasmodium falciparum* 3D7 parasites was obtained by enriching trophozoite/schizont-infected red blood cells by centrifugation through 60% Percoll, incubating the enriched cells with fresh red blood cells in culture medium for 8 hours, followed by sorbitol treatment. After a further overnight incubation, the parasites had reached the trophozoite stage (0 hours image above) and ATP levels were measured every 2 hours over an 8 hour period. Representative images of Giemsa-stained thin-smears of parasitized red blood cells at the various time points of trophozoite development used in the ATP time-course assay are shown below the graph. Click here for file
