# Supplementary material for: ATP and luciferase assays to determine the rate of drug action in in vitro cultures of Plasmodium falciparum
Source: Malar J. 2012 Nov 7;11:369. doi: 10.1186/1475-2875-11-369 (PMC3505462; doi:10.1186/1475-2875-11-369)
Supplement: Additional file 1 — Figure A1. Correlation of ATP luminescence signals with parasite numbers and stages. A. Aliquots were removed from a trophozoite-stage culture and the parasites isolated. Serial dilutions of the parasites were performed in PBS and ATP levels measured. Parasite numbers were calculated from the percentage parasitaemia and red blood cell concentration (determined with a haemocytometer) of the original culture. Three samples were processed in parallel. Note that the lowest parasite number (1.23x10^5^) corresponds to approximately 4μl of a 2% parasitaemia, 5% haematocrit culture (or 0.2μl packed red blood cells) and produced an average luminescence reading of 7030. B. Ring-stage parasites were isolated from three 0.5ml aliquots of a sorbitol synchronized 5% haematocrit 10% parasitaemia culture and snap-frozen in liquid nitrogen. After 24h, additional 0.5ml aliquots were removed from the same culture and used to prepare frozen trophozoite-stage parasites. After thawing, ATP luminescence signals in the samples were determined and compared to background signals obtained with aliquots of uninfected red blood cell cultures processed in parallel. The trophozoite samples produced a mean luminescence reading of 354054, compared 13121 for rings and 993 for uninfected red blood cells (note the log scale of the Y-axis). [file 1475-2875-11-369-S1.pdf]

Additional file 1

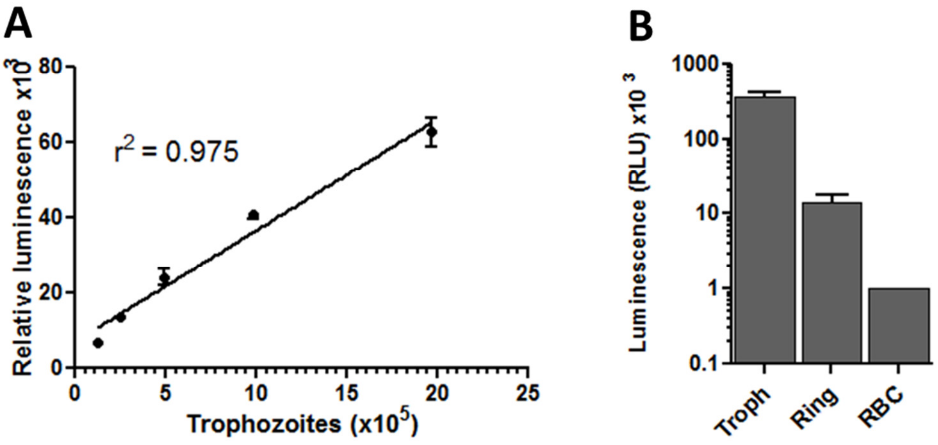

Figure A1: Correlation of ATP luminescence signals with parasite numbers and stages. A. Aliquots were removed from a trophozoite-stage culture and the parasites isolated. Serial dilutions of the parasites were performed in PBS and ATP levels measured. Parasite numbers were calculated from the percentage parasitaemia and red blood cell concentration (determined with a haemocytometer) of the original culture. Three samples were processed in parallel. Note that the lowest parasite number ($1.23x10^5$) corresponds to approximately 4µl of a 2% parasitaemia, 5% haematocrit culture (or 0.2µl packed red blood cells) and produced an average luminescence reading of 7030. **B.** Ring-stage parasites were isolated from three 0.5ml aliquots of a sorbitol synchronized 5% haematocrit 10% parasitaemia culture and snap-frozen in liquid nitrogen. After 24h, additional 0.5ml aliquots were removed from the same culture and used to prepare frozen trophozoite-stage parasites. After thawing, ATP luminescence signals in the samples were determined and compared to background signals obtained with aliquots of uninfected red blood cell cultures processed in parallel. The trophozoite samples produced a mean luminescence reading of 354054, compared 13121 for rings and 993 for uninfected red blood cells (note the log scale of the Y-axis).
