# Supplementary material for: ATP and luciferase assays to determine the rate of drug action in in vitro cultures of Plasmodium falciparum
Source: Malar J. 2012 Nov 7;11:369. doi: 10.1186/1475-2875-11-369 (PMC3505462; doi:10.1186/1475-2875-11-369)
Supplement: Additional file 2 — Table 1. IC50 values of compounds used in this study. IC50 values were obtained by incubating P. falciparum 3D7 cultures with serial dilutions of the test compounds for 48 hours and assessing parasite viability using the parasite lactate dehydrogenase (pLDH) assay. Values are shown as averages ± standard deviation for three independent determinations. IC50 values for lactacystin and MG-132 were determined on a single occasion against luciferase-expressing parasites. [file 1475-2875-11-369-S2.pdf]

Additional file 2

Table 1: IC_{50} values of compounds used in this study. IC_{50} values were obtained by incubating *P. falciparum* 3D7 cultures with serial dilutions of the test compounds for 48 hours and assessing parasite viability using the parasite lactate dehydrogenase (pLDH) assay. Values are shown as averages ± standard deviation for three independent determinations. IC_{50} values for lactacystin and MG-132 were determined on a single occasion against luciferase-expressing parasites.

**Compound**	**IC50**
Chloroquine	15.9 nM ±4.8
DFMO	1.9 mM ±0.6
Mefloquine	16.2 nM ±3.6
Artemisinin	20.5 nM ±6.5
Gramicidin	0.022 nM ±0.001
Ritonavir	14.2 µM ±0.7
Lactacystin	0.84 µM
MG-132	58.5 nM
