# Supplementary material for: ATP and luciferase assays to determine the rate of drug action in in vitro cultures of Plasmodium falciparum
Source: Malar J. 2012 Nov 7;11:369. doi: 10.1186/1475-2875-11-369 (PMC3505462; doi:10.1186/1475-2875-11-369)
Supplement: Additional file 3 — Figure A2. ATP changes during trophozoite development. A tightly synchronized culture of Plasmodium falciparum 3D7 parasites was obtained by enriching trophozoite/schizont-infected red blood cells by centrifugation through 60% Percoll, incubating the enriched cells with fresh red blood cells in culture medium for 8 hours, followed by sorbitol treatment. After a further overnight incubation, the parasites had reached the trophozoite stage (0 hours image above) and ATP levels were measured every 2 hours over an 8 hour period. Representative images of Giemsa-stained thin-smears of parasitized red blood cells at the various time points of trophozoite development used in the ATP time-course assay are shown below the graph. [file 1475-2875-11-369-S3.pdf]

Additional file 3

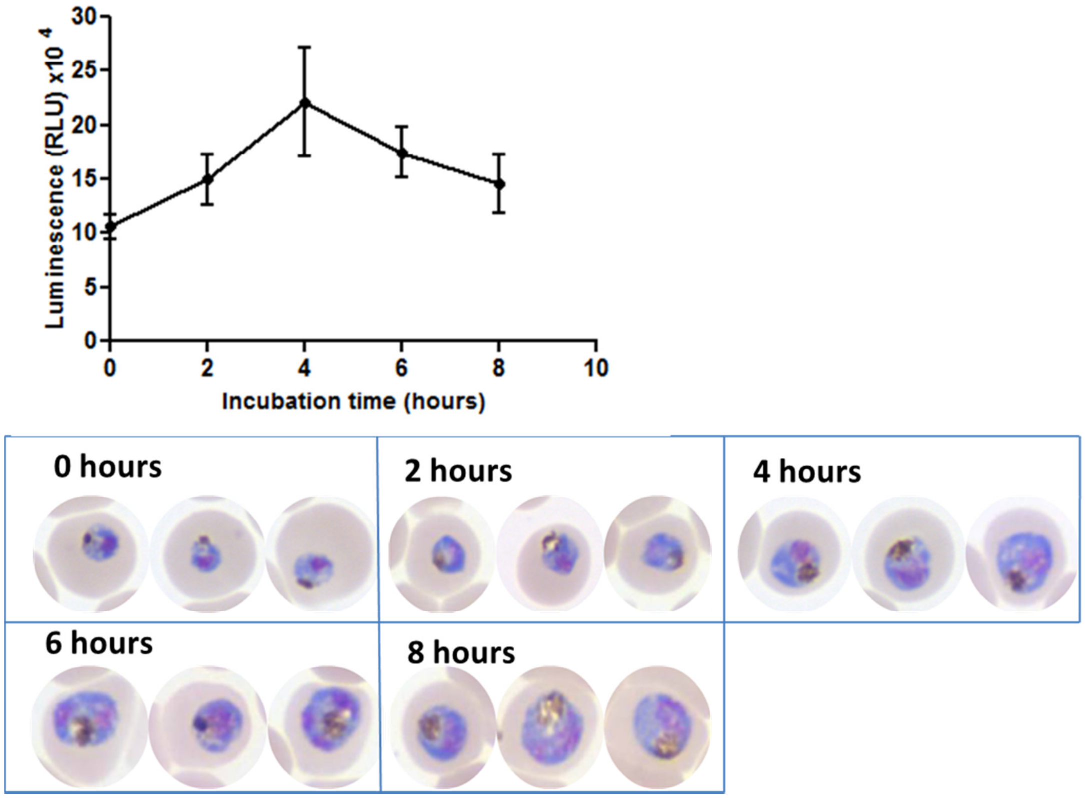

Figure A2: ATP changes during trophozoite development. A tightly synchronized culture of *Plasmodium falciparum* 3D7 parasites was obtained by enriching trophozoite/schizont-infected red blood cells by centrifugation through 60% Percoll, incubating the enriched cells with fresh red blood cells in culture medium for 8 hours, followed by sorbitol treatment. After a further overnight incubation, the parasites had reached the trophozoite stage (0 hours image above) and ATP levels were measured every 2 hours over an 8 hour period. Representative images of Giemsa-stained thin-smears of parasitized red blood cells at the various time points of trophozoite development used in the ATP time-course assay are shown below the graph.
